# Rational synthesis of microporous carbons for enhanced post-combustion CO_2_ capture *via* non-hydroxide activation of air carbonised biomass†

**DOI:** 10.1039/d2ra02661a

**Published:** 2022-07-12

**Authors:** Afnan Altwala, Robert Mokaya

**Affiliations:** School of Chemistry, University of Nottingham University Park Nottingham NG7 2RD UK r.mokaya@nottingham.ac.uk; Department of Chemistry, College of Science Al-Zulfi, Majmaah University Al-Majmaah 11952 Saudi Arabia

## Abstract

This work explores the use of a less corrosive activating agent, potassium oxalate (PO), in combination with difficult to activate carbonaceous matter for the preparation of activated carbons. The design of the study allowed a fuller understanding of the workings of PO compared to hydroxide (KOH) activation, and also optimised the preparation of highly microporous carbons with exceptional CO_2_ storage capacity under low pressure (≤1 bar) conditions at ambient temperature. The PO activated carbons have a surface area of up to 1760 m^2^ g^−1^ and are highly microporous with virtually all of the surface area arising from micropores. The porosity of the PO activated carbons can be readily tailored towards having pores of size 6–8 Å, which are highly suited for CO_2_ storage at low pressure (*i.e.*, post-combustion capture). At 25 °C, the PO activated carbons can store up to 1.8 and 5.0 mmol g^−1^ of CO_2_ at 0.15 bar and 1 bar, respectively. On the other hand, KOH activated carbons reach a higher surface area of up to 2700 m^2^ g^−1^, and store up to 1.0 and 4.0 mmol g^−1^ of CO_2_. This work demonstrates that PO may be used as a mild, less corrosive and less toxic activating agent for the rational and targeted synthesis of biomass-derived activated carbons with tailored porosity. The targeted synthesis may be aided by careful selection of the biomass starting material as guided by the O/C ratio of the biomass.

## Abstract 2

Rational combination of a mild activating agent (potassium oxalate) and air carbonised biomass, which is resistant to activation, yields highly microporous carbons with enhanced post-combustion CO_2_ uptake.

## Graphical abstract

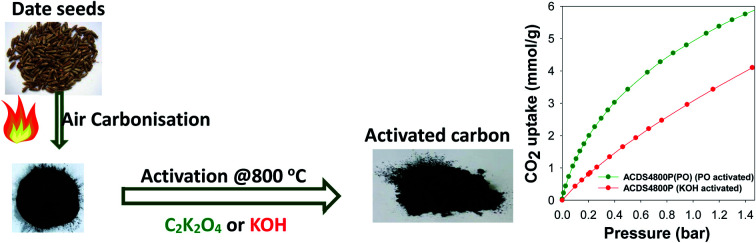

## 1. Introduction

The concentration of CO_2_ in the atmosphere is rapidly increasing. This increase is a significant global challenge as CO_2_ is a greenhouse gas in terms of contributing to global warming. Carbon capture and storage (CCS) is among the few remaining options for halting global warming.^1–5^ Activated porous carbons have long been used in environmental remediation applications, including removal of water pollutants, sewage treatment, air purification, and more recently have attracted attention in energy storage applications.^6–11^ As a result of their suitable chemical and thermal stability, and high and controllable porosity, activated carbons are amongst the most promising porous materials for CO_2_ capture and storage.^12–21^ In recent years, new ways of preparing porous carbons with properties directly targeted at energy-related applications have been a focus of intense research efforts.^22–33^

The most frequently used chemical activating agents for the preparation of activated carbons are inorganic acids (HCl, H_2_SO_4_, and H_3_PO_4_), alkali hydroxides (KOH, NaOH), ZnCl_2_, and K_2_CO_3_.^14–16^ KOH is one of the best-studied activating agent, as it can generate activated carbon with defined pore size distribution and high surface area from a wide range of carbon starting materials (*i.e.*, precursors).^27–31^ However, the use of KOH has disadvantages associated with its toxic and corrosive nature. Milder activating agents that yield carbons with comparable or superior textural properties compared to KOH activation are therefore a key research target.^34–44^ In particular, in the search for activating agents beyond hydroxides, the use of potassium oxalate (PO) as activator for a range of precursors has been explored.^34–38,40–44^ Previous studies on PO activated carbons have shown that, in general, carbons with low to medium surface area (typically <1500 m^2^ g^−1^) are obtained at activation temperature below 800 °C.^34–38,44^

The elemental composition and pore structure of activated carbons depend on the nature of the precursor(s) and the activation parameters. For thermal activation with an activating agent, the parameters include duration of activation, type and flow rate of the inert gas used to create a non-oxidising atmosphere, heating ramp rate and, most importantly, the temperature and amount of activating agent. Additionally, as demonstrated recently,^30,45^ the O/C ratio of any carbonaceous matter utilised as precursor is an essential factor in determining the porosity of KOH-activated carbons, with a high O/C ratio favouring greater surface area and pore volume. This means that in addition to activation parameters, the properties of porous carbons can also be influenced by choice of precursors, allowing for better targeting and tailoring of porosity towards specific applications.^30,45^

Biomass sources are widely available as a renewable resource, are generally affordable and may be considered as being ‘carbon neutral’. We have previously shown that date seeds (*Phoenix dactylifera*), once air-carbonised in a process that is simpler than conventional hydrothermal carbonisation (HTC)^46–48^ or pyrolysis,^49–52^ generate carbonaceous matter with low O/C ratio that may be used as precursor to generate KOH-activated carbons with properties suited for methane storage.^45^ We demonstrated that the low O/C ratio of air-carbonised date seed plays a key role in determining the porosity of the resulting KOH-activated carbons.^45^ In the present study, milder PO activation of air carbonised date seed was performed and compared to KOH activation. This work focussed on exploration of the possibility of generating highly microporous activated carbons by combining a mild activating agent (PO) with a precursor that has low O/C ratio and is therefore resistant to activation. The expectation was that this will limit the level of activation (compared to the use of KOH) and thus hinder the formation of mesopores, while at the same time offering high yields of activated carbons that only possess micropores. Given the expectation of highly microporous carbons, this study targeted their application towards the uptake of CO_2_ at low pressures that mimic post-combustion capture from the flue gas streams of fossil fuel power stations.

## 2. Experimental section

### 2.1 Material preparation

After thorough washing with water and drying, date seed (5 g) were placed in an alumina boat and heated in a horizontal tube furnace, at a heating ramp rate of 10 °C min^−1^, to 400 °C under a nitrogen atmosphere. Once at 400 °C, the date seed was exposed to a flow of air for 5–10 min, after which the furnace was left to cool under a flow of nitrogen gas. The resulting carbonaceous matter was designated as ACDS (*i.e.*, air carbonised date seed) carbon. For activation, the ACDS carbon was mixed with the required amount of potassium oxalate (PO) at predetermined PO/ACDS carbon ratios in an agate mortar. The PO + ACDS carbon mixture was placed in an alumina boat and heated in a furnace to 700 or 800 °C at a heating ramp rate of 3 °C min^−1^ under an atmosphere of nitrogen gas and held at the final temperature for 1 h, after which the sample was allowed to cool under a flow of nitrogen. The resulting activated samples were then washed by stirring in 20% aqueous HCl at room temperature, and then repeatedly with deionised water until the filtrate was neutral (pH ∼ 7). The samples were then dried in an oven at 120 °C, and designated as ACDS*xT*(PO), where *x* is the PO/ACDS carbon ratio, *T* is the activation temperature (in °C) and PO indicates the use of potassium oxalate as activating agent. Thus, a carbon activated at a PO/ACDS carbon ratio of 2 and at 800 °C is designated as ACDS2800(PO) or ACDS2800P(PO), where P indicates that the PO + ACDS mixture was compacted before the activation step (*i.e.*, compactivation). The compaction into pellets prior to activation was performed in a 1.3 cm die for *ca.* 5 min at 740 MPa.

Carbons activated at a KOH/ACDS carbon ratio of 2 or 4 and at 700 or 800 °C were also explored for CO_2_ uptake in order to make a clear comparison between KOH and PO activation. A carbon activated at a KOH/ACDS carbon ratio of 2 and at 800 °C is designated as ACDS2800 for a powder sample and ACDS2800P for a compactivated sample.

### 2.2 Material characterisation

Thermogravimetric analysis (TGA) was performed using a TA Instruments Discovery analyser or TA Instruments SDT Q600 analyser under flowing air conditions (100 mL min^−1^). A PANalytical X'Pert PRO diffractometer was used to perform powder XRD analysis using Cu-Kα light source (40 kV, 40 mA) with step size of 0.02° and 50 s time step. Elemental, CHN, analysis was performed on an Exeter Analytical CE-440 Elemental Analyser. Nitrogen sorption (at −196 °C) with a Micromeritics 3FLEX sorptometer was used for porosity analysis and to determine textural properties. Prior to analysis the carbon samples were degassed under vacuum at 200 °C for 12 h. Surface area was calculated using the Brunauer–Emmett–Teller (BET) method applied to adsorption data in the relative pressure (*P*/*P*_0_) range of 0.02–0.22, and pore volume was estimated from the total nitrogen uptake at close to saturation pressure (*P*/*P*_0_ ≈ 0.99). The micropore surface area and micropore volume were determined *via t*-plot analysis. The pore size distribution was determined using non-local density functional theory (NL-DFT) applied to nitrogen adsorption data. Scanning electron microscopy (SEM) images were recorded using an FEI Quanta200 microscope, operating at a 5 kV accelerating voltage. Transmission electron microscopy (TEM) images were obtained using a JEOL 2100F instrument operating at 200 kV equipped with a Gatan Orius CCD for imaging. Prior to analysis, the carbon samples were suspended in distilled water and dispersed onto lacey carbon support films.

### 2.3 CO_2_ uptake measurements

CO_2_ uptake was determined using a Hiden Isochema XEMIS instrument at 25 °C and pressure of up to 20 bar. The carbons were outgassed for 3 h under vacuum at 240 °C prior to performing the CO_2_ uptake measurements. The measurements directly determined the excess CO_2_ uptake.

## 3. Results and discussion

### 3.1 Structural ordering, morphology and thermal stability of carbons

Following flash air-carbonisation, the carbon content increased from 49 wt% for raw date seeds to 78.5 wt% for ACDS carbon, whereas the H content decreased from 7 wt% to 4 wt%. This was accompanied by a significant decrease in the apparent O content from 42.4 wt% (raw date seed) to 16.3 wt% for ACDS carbon. Table 1 summarises the carbon yields of the PO activated carbons with the yield being in the range of 58% to 64% for samples activated at 700 °C, and 52% to 60% for activation at 800 °C. This compares to yields of between 46% and 60% for KOH activation of ACDS carbon (ESI Table S1†) meaning that, in general, the yield achieved from PO activation is higher. We also compared the yield of PO activation of ACDS carbon with PO activation of hydrochar derived from sawdust *via* hydrothermal carbonisation.^34^ Under similar activation conditions (*i.e.*, PO/hydrochar ratio 2 or 4 and activation temperature of 700 and 800), the yield from the hydrochar was in the range of 36% to 41%, which is significantly lower than for PO activation of ACDS carbon.^34^ This comparison clearly demonstrates the benefits, in terms of greater yield, of utilizing air-carbonised date seed as a precursor; the genesis of this benefit arises from the nature of the ACDS carbon as occasioned by its low O/C ratio and associated resistance to activation.^45^

**Table 1:** Yield and elemental composition of PO activated carbons derived from ACDS carbon

Sample	Yield [wt%]	C [%]	H [%]	N [%]	O [%]
Date seed		49.0	7.0	1.6	42.4
ACDS carbon		78.5	4.0	1.2	16.3
ACDS2700(PO)	58	80.6	1.0	2.6	15.8
ACDS2800(PO)	52	83.0	0.1	0.2	16.7
ACDS4700(PO)	62	80.0	1.2	1.8	17.0
ACDS4800(PO)	60	84.0	0	0.2	15.8
ACDS2700P(PO)	60	79.3	1.0	1.0	18.7
ACDS2800P(PO)	56	87.0	0.1	0.3	12.6
ACDS4700P(PO)	64	79.5	0.2	0.1	20.2
ACDS4800P(PO)	58	82.5	0	0.3	17.2

The elemental C content of the PO activated carbons increases from 78.5 wt% for the ACDS carbon to as high as 87.0 wt% (ACDS2800P(PO)), while the content of both N and H reduces to trace or nil amounts for some of the activated samples. KOH activated carbons (Table S1†) show a similar trend in CHN content with the highest C content reaching 90 wt% (ACDS2800). For both PO and KOH, therefore, the activation of ACDS carbon results in an increase in the C content, with the increase being generally greater at higher activation temperature.

The TGA curves of PO activated carbons (ESI Fig. S1†) show a small initial mass loss below 100 °C, which is ascribed to evaporation of residual moisture, followed by the main mass loss step due to carbon burn-off. The curves indicate that the activated carbons are stable up to at least 400 °C, with carbon burn-off occurring between 400 °C and 660 °C. Samples activated at 800 °C appear to exhibit greater thermal stability. The wide burn-off temperature range (400 to 660 °C) is consistent with the amorphous (*i.e.*, non-graphitic) nature of the carbons. The activated carbons display nil or <2 wt% residual mass, suggesting that they may be considered as being essentially fully carbonaceous with only trace or nil inorganic matter. This is confirmed by XRD patterns (ESI Fig. S2†) that show broad peaks at 2-theta of 22° and 44° and which corresponds to positions where the (002) and (100) diffractions are expected to arise from graphene stacks. SEM and TEM were used to ascertain the morphology and pore ordering of the carbon materials. SEM images (ESI Fig. S3†) revealed morphology that is dominated by honeycomb structures consistent with previous reports.^24–28^ On the other hand, TEM (ESI Fig. S4 and S5†) revealed wormhole-type pore channels that are typical for activated carbons. The TEM images show no significant evidence of the presence of graphitic domains, which is in agreement with previous studies.^20,21,24–28^

### 3.2 Textural properties and porosity

**Fig. 1:**
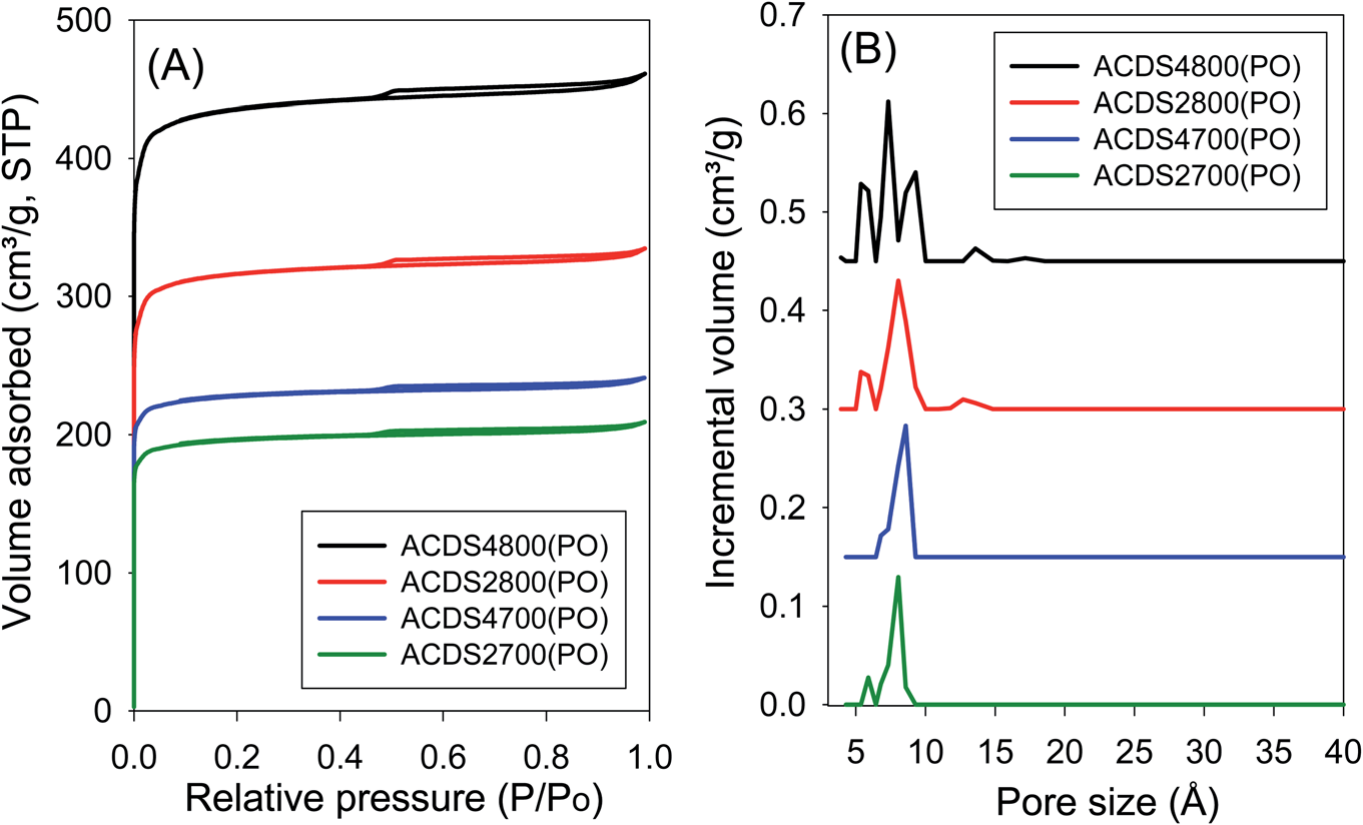
Nitrogen sorption isotherms (A) and pore size distribution curves (B) of PO activated carbons derived from powdered ACDS carbon.

**Fig. 2:**
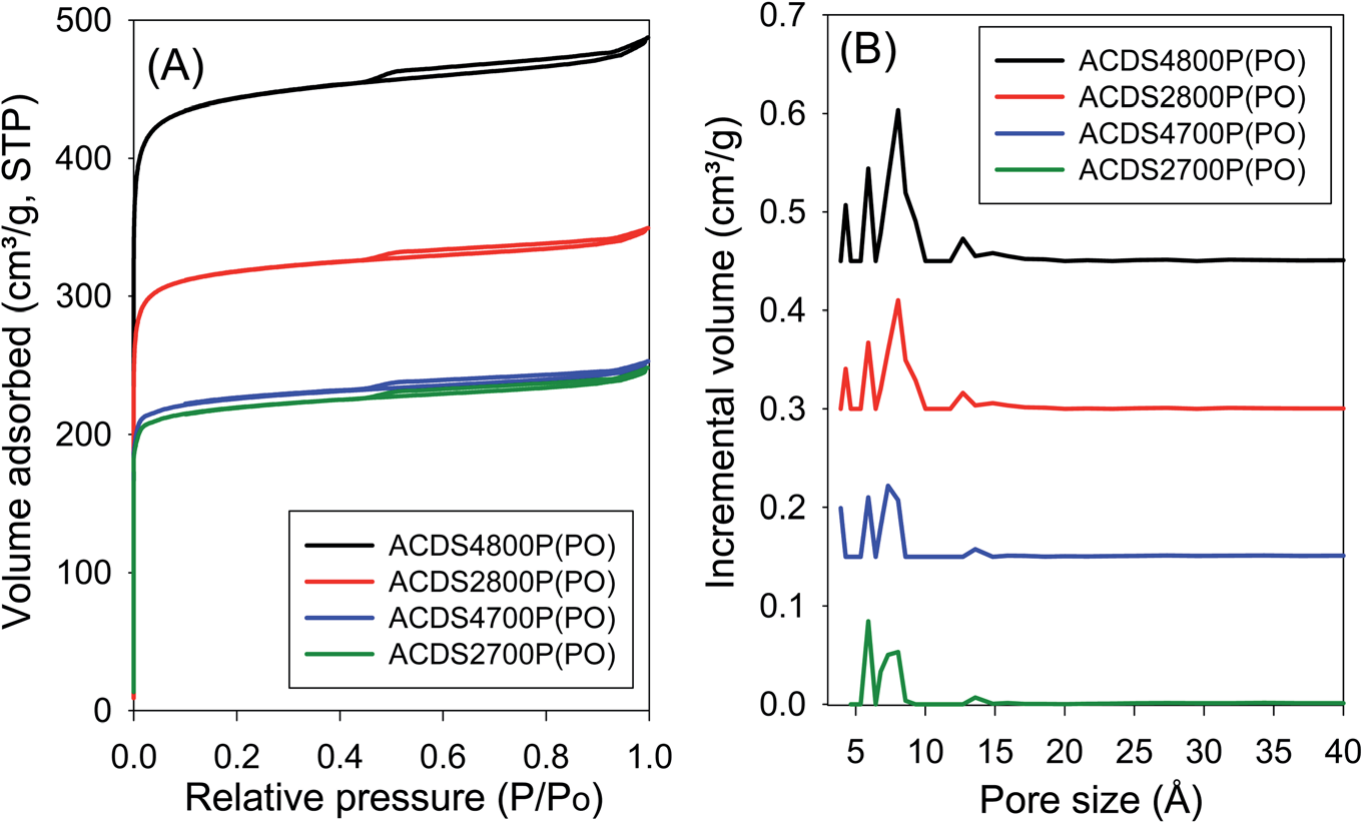
Nitrogen sorption isotherms (A) and pore size distribution curves (B) of PO compactivated carbons derived from compacted ACDS carbon.

The ACDS carbon was found to be nearly non-porous, with a surface area of 2.5 m^2^ g^−1^ and pore volume of 0.004 cm^3^ g^−1^. Fig. 1 and 2 show, respectively, the nitrogen sorption isotherms and corresponding pore size distribution (PSD) curves of carbons activated as powders or pellets (compactivation). All the samples, regardless of activation conditions (powder or pellet, amount of PO or temperature) exhibit type I isotherms indicating that the carbons are microporous. The microporous nature of the carbons is such that most nitrogen sorption occurs at a low relative pressure (*P*/*P*_0_ < 0.1), with hardly any further adsorption at *P*/*P*_0_ above 0.1. Pelletization (compaction) before activation has little effect on the porosity generated. This is related to the fact that the key determinant in the porosity of PO activated carbons is the activation temperature rather than the amount of PO.^34,44^ Compactivation has previously been shown to improve the efficiency of KOH as an activating agent but such improvement does not appear to apply to PO at PO/ACDS carbon ratio of 2 and 4. In a sense, the amount of PO can be considered to be already tending towards being ‘in excess’ at PO/ACDS carbon ratio of 2 and thus any process modification to improve the action of PO will have little effect. On the other hand, as shown in Fig. 1 and 2, the amount of nitrogen adsorbed is higher for samples activated at 800 °C, which is an indication of greater porosity. It is, however, interesting to note that despite the increase in amount of nitrogen adsorbed as activation temperature rises from 700 to 800 °C, there is little change in the shape of the isotherms as all the carbons exhibit isotherms typical of microporous materials with a sharp adsorption knee. Sharp adsorption knees are associated with the absence of pores of size larger than the micropore range (<20 Å) as confirmed by the PSD curves (Fig. 1 and 2).

Unlike for PO activation, the isotherms of KOH activated carbons (ESI Fig. S6 and S7†) show that some samples have a significant proportion of supermicropores (*i.e.*, pore channels of size 7–20 Å) and some small mesopores.^45^ Clearly, KOH activation generates a greater proportion of larger pores compared to PO activation, and the proportion of these larger pores (*i.e.*, mesopores) is higher at KOH/ACDS carbon ratio of 4. Indeed, KOH activation, especially at 800 °C and/or KOH/ACDS ratio of 4, generates carbons (ESI Fig. S8†) with a relatively wide pore size range that extends up to 25 Å unlike for PO activation where there are hardly any pores wider than 10 Å (Fig. 1 and 2).

The textural properties of PO activated carbons are shown in Table 2. The total surface area is in the range of 790–1767 m^2^ g^−1^ with pore volume of between 0.31 and 0.75 cm^3^ g^−1^, both of which are low to moderate compared to other porous carbons.^14–32^ The surface area and pore volume increase with the activation temperature but are unaffected by the amount of PO. The low to moderate porosity observed is in keeping with both the mild nature of PO as activator, and the resistant to activation nature of ACDS carbon occasioned by its low O/C ratio. The effect of combining a mild activator with a hard to activate precursor is also amplified by the extremely high level of microporosity observed; the proportion of microporosity for the PO activated carbons is 94–97% of surface area and 82–94% of pore volume, meaning that virtually all of the surface area arises from micropores. This is despite the fact that the magnitude of surface area more than doubles as the activation temperature rises from 700 °C to 800 °C. This is a noteworthy finding given that for most activated carbons, in general, an increase in overall porosity arising from more severe levels of activation usually results in a diminution in microporosity; indeed such a trend is observed for KOH activated carbons (ESI Table S2†). That the high microporosity of PO activated carbons is in part due to use of a mild activating agent is amplified by the fact that KOH activated carbons (Table S2†) present lower levels of micropore surface area (70–90%) and micropore volume (63–88%). Thus, unlike PO activation, the microporosity of KOH activated carbons significantly reduces at harsher levels of activation such that micropores contribute only 70% and 63% of surface area and pore volume, respectively, for KOH activated ACDS4800 (Table S2†) compared to (Table 2) 97% of surface area and 93% of pore volume for the equivalent PO activated sample (ACDS4800(PO)).

**Table 2:** Textural properties of PO activated and compactivated carbons derived from ACDS carbon

Sample	Surface area (m^2^ g^−1^)	Micropore surface area^a^ (m^2^ g^−1^)	Pore volume (cm^3^ g^−1^)	Micropore volume^b^ (cm^3^ g^−1^)	Pore size^c^ (Å)
ACDS2700(PO)	790	767 (97)	0.32	0.30 (94)	4/6/8
ACDS4700(PO)	918	893 (97)	0.37	0.34 (92)	4/6/8
ACDS2800(PO)	1261	1223 (97)	0.51	0.47 (92)	6/8
ACDS4800(PO)	1748	1696 (97)	0.71	0.66 (93)	6/8/9
ACDS2700P(PO)	881	825 (94)	0.38	0.31 (82)	6/8
ACDS4700P(PO)	908	855 (94)	0.38	0.32 (84)	6/8
ACDS2800P(PO)	1267	1202 (95)	0.54	0.47 (87)	6/8
ACDS4800P(PO)	1767	1676 (95)	0.75	0.65 (87)	4/6/8

a The values in the parenthesis refer to % micropore surface area.

b The values in the parenthesis refer to % micropore volume.

c Pore size maxima from the pore size distribution curves.

Interestingly, there does not appear to be any significant difference between the overall porosity of compacted and powder samples. This is in contrast to KOH activation (Table S2†) where compactivation always results in an increase in surface area and pore volume, and decrease in microporosity. For PO activation, the only effect of compaction is a slight reduction in the proportion of pore volume arising from micropores. However, based on the PSD curves (Fig. 1 and 2) and the pore size maxima (Table 2), this slight decrease in proportion of micropore volume is not due to the formation of larger pores but may be related to variations in particle packing that give rise to interparticle voids. This suggestion is consistent with the fact that it is only the total pore volume that is higher in compactivated carbons; the surface area (both total and micropore) and micropore volume remain unchanged. The assumption here is that interparticle voids, being much larger spaces, would mainly contribute to the total pore volume.

The differences in porosity described above are related to the action of KOH and PO. For KOH, the hydroxide reacts directly with C to generate carbonates, meaning that activation (C etching) can occur even at low temperatures. On the other hand, PO activation occurs *via*, firstly, decomposition of the oxalate to the carbonate, which is then followed by gasification reactions at high temperatures that constitute the activation steps. The PO activation mechanism is as follows;^34–38,40–44^ at 500–600 °C, PO decomposition occurs (K_2_C_2_O_4_ → K_2_CO_3_ + CO). This is followed by a reaction, at 700 °C and above, between C within the precursor and the generated carbonate, which results in the etching of C atoms (*i.e.*, pore formation) according to the reaction K_2_CO_3_ + 2C → 2K + 3CO. Moreover, the carbonate can also decompose (K_2_CO_3_ → K_2_O + CO_2_), generating CO_2_ gas that can cause pore formation (at ≥700 °C) *via* gasification (C + CO_2_ → 2CO) of the ACDS carbon. Activation *via* oxalate decomposition explains the mild nature of PO compared to the more direct action of KOH with respect to interaction with the precursor. Thus using potassium oxalate as an activating agent allows for porosity control by simply changing the activation temperature, and benefits from being a less corrosive and less toxic activating agent than KOH. For the present activation of the ACDS carbon, the overall trends in porosity that emerge from the PO and KOH comparison are that, at temperatures of 700 to 800 °C, the former (PO) generates carbons with a surface area of up to *ca.* 1800 m^2^ g^−1^, whereas KOH activation achieves carbons with a surface area of up to 2700 m^2^ g^−1^. However, carbons with a higher proportion of micropore surface area, which is preferred for CO_2_ uptake at low pressure, are generated with PO.

### 3.3 CO_2_ uptake

**Fig. 3:**
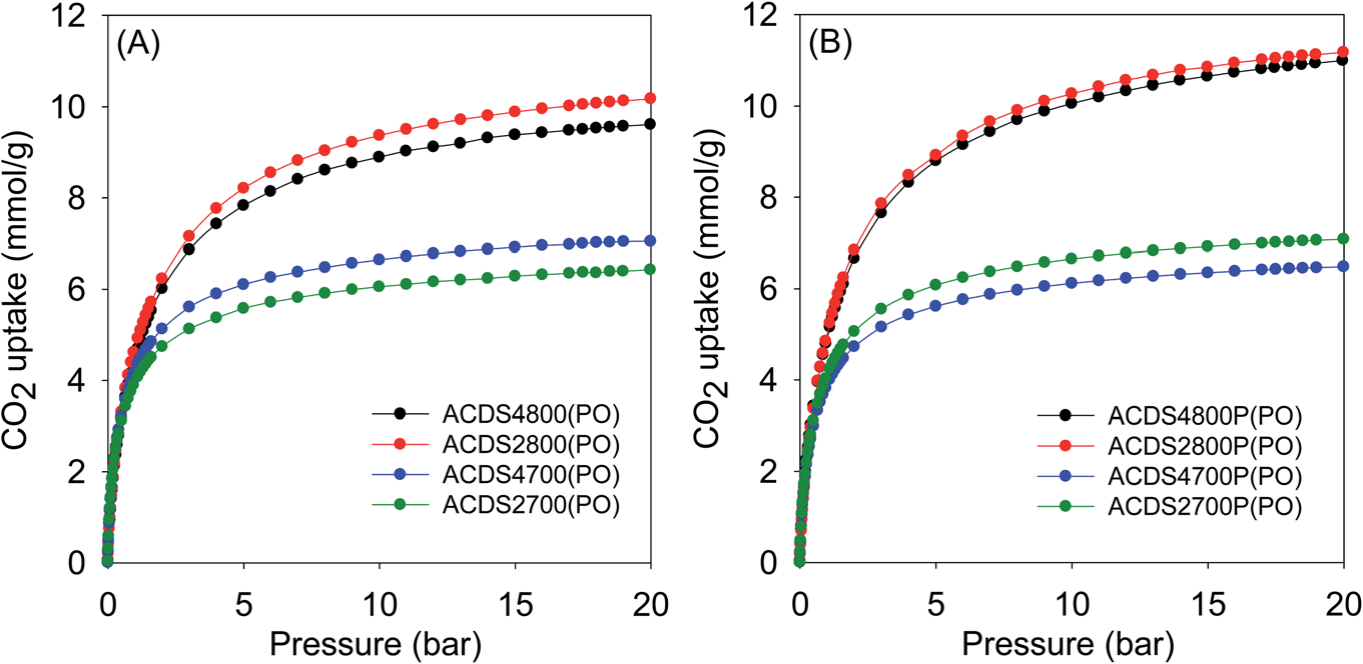
CO_2_ uptake at 25 °C of PO activated carbons derived from (A) powdered or (B) compacted ACDS carbon.

**Fig. 4:**
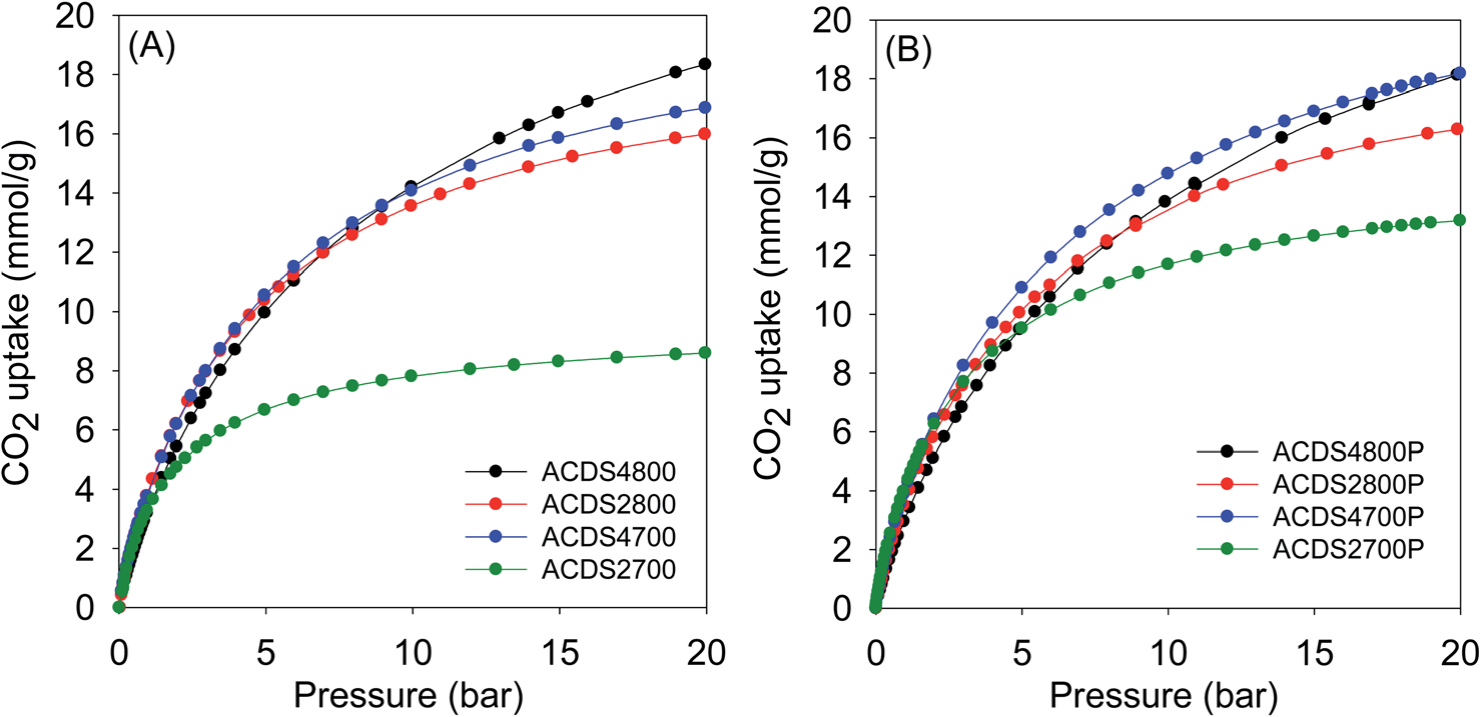
CO_2_ uptake at 25 °C of KOH activated carbons derived from (A) powdered or (B) compacted ACDS carbon.

The textural properties of the present carbons, and in particular their very high levels of microporosity, were specifically targeted towards suitability for CO_2_ storage at low pressures that mimic post-combustion capture from flue gas streams of fossil fuel power stations. Fig. 3 and 4 show the CO_2_ uptake isotherms for PO and KOH activated carbons, respectively, and Tables 3 and 4 summarize the storage capacity (mmol g^−1^) at 0.15, 1 and 20 bar. The PO activated carbons have very high uptake of between 1.5 and 1.8 mmol g^−1^ at 0.15 bar. This uptake pressure was chosen as an indicator of the storage capacity from a flue gas stream (at 1 bar) that typically contains *ca.* 15% CO_2_. The uptake capacity places the PO carbons at the top end of porous materials given that for most previously reported activated carbons, the uptake at 0.15 bar is typically lower than 1 mmol g^−1^.^19–40,53^ The narrow range of the uptake capacity at 0.15 bar is a reflection of the similarity in pore size of the PO activated carbons regardless of their overall surface area and pore volume. Indeed, the magnitude of the surface area and pore volume does not have any effect on the uptake. As shown in Table 4, the uptake of KOH activated carbons is lower at 0.6–1.0 mmol g^−1^, which is a reflection of their lower levels of microporosity and presence of wider micropores and small mesopores (Fig. S6–S8 and Table S2†). Thus use of PO as activator for ACDS carbon allows for a more than doubling of the CO_2_ uptake at 0.15 bar.

**Table 3:** CO_2_ uptake at 25 °C of PO activated and compactivated carbons derived from ACDS carbon

Sample	CO_2_ uptake (mmol g^−1^)		
	0.15 bar	1 bar	20 bar
ACDS2700(PO)	1.8	4.0	6.4
ACDS4700(PO)	1.8	4.2	7.0
ACDS2800(PO)	1.6	4.8	10.2
ACDS4800(PO)	1.5	4.6	9.6
ACDS2700P(PO)	1.6	4.1	7.1
ACDS4700P(PO)	1.6	4.0	6.5
ACDS2800P(PO)	1.5	5.0	11.0
ACDS4800P(PO)	1.6	4.9	11.0

**Table 4:** CO_2_ uptake at 25 °C of KOH activated and compactivated carbons derived from ACDS carbon

Sample	CO_2_ uptake (mmol g^−1^)		
	0.15 bar	1 bar	20 bar
ACDS2700	0.7	3.4	8.6
ACDS4700	0.9	3.9	16.9
ACDS2800	0.7	3.9	16.0
ACDS4800	0.7	3.3	18.3
ACDS2700P	1.0	3.7	15.0
ACDS4700P	0.9	4.0	18.2
ACDS2800P	0.7	3.7	16.3
ACDS4800P	0.6	3.1	18.1

At 1 bar (Fig. 3 and S9†), the PO activated carbons store between 4.0 and 5.0 mmol g^−1^ of CO_2_, which compares well with the best reported values for carbon materials.^12–25,53–69^ Carbons activated at 800 °C, and which have higher overall surface area and pore volume, have higher CO_2_ uptake. This is to be expected because the increase in porosity for samples activated at 800 °C is not at the expense of microporosity, *i.e.*, there is simply more of the same surface available to CO_2_ adsorption. This contrasts with what has typically been reported for activated carbons where more severe activation leads to greater porosity and a decrease in microporosity with the consequence that CO_2_ uptake at 1 bar is unchanged or reduces.^12–25^ Indeed, this is what is observed for KOH activated carbons as shown in Table 4. The uptake of KOH activated carbons at 1 bar (ESI Fig. S10†), at 3.1–4.0 mmol g^−1^, is lower than that of PO activated samples (4.0–5.0 mmol g^−1^). This is despite the KOH activated carbons having higher surface area and pore volume, which emphasizes the absence of a link between low pressure CO_2_ uptake and total porosity. The lower uptake (at ≤1 bar) of the KOH carbons is due to their wider pores compared to PO activated equivalents (Fig. S8†). The situation is reversed at 20 bar where the CO_2_ storage capacity of KOH activated carbons is much higher due to greater dependence of uptake on the total surface area. Overall, therefore, the gravimetric CO_2_ storage capacity of PO activated carbons under post combustion capture conditions (≤1 bar) outperforms that of analogous KOH activated samples. At 0.15 bar, the uptake of PO carbons is more than double that of KOH samples, while at 1 bar the improvement is as high as 60% for some samples. Thus despite the PO carbons being prepared with a milder and much easier to handle activating agent, they are very attractive for CO_2_ capture and storage and compare favorably with current benchmark porous carbons.^12–25,53–76^

Moreover, according to the uptake data in Table 3, there is no clear correlation between the amount of PO used in activation and the CO_2_ uptake of the samples. Therefore, changing the PO/ACDS ratio between 2 to 4 does not significantly affect the CO_2_ uptake. For instance, the CO_2_ uptake at 1 bar is *ca.* 5.0 mmol g^−1^ for both sample ACDS2800P(PO) and ACDS4800P(PO). Additionally, the CO_2_ uptake at 20 bar is 11 mmol g^−1^ for both sample ACDS2800P(PO) and ACDS4800P(PO) and is very similar (9.5–10.5 mmol g^−1^) for sample ACDS2800(PO) and ACDS4800(PO). This means that there is no need to use the higher amount of PO as a means to optimising the CO_2_ uptake. Use of a lower amount of PO is attractive as it reduces the costs of activation thus making the process more affordable and sustainable. This is in addition to the milder nature (in terms of corrosion) of PO activation, which will translate to lower costs with respect to equipment maintenance. In contrast, the activation temperature has an essential role in determining the CO_2_ uptake. Higher activation temperature does in some cases translate to greater CO_2_ uptake especially at pressures above 1 bar. In a sense, the activation temperature can be used to tailor the porosity of the PO activated carbons so as to target optimised CO_2_ uptake at low or high pressure. In this regard, temperature variations combined with a meagre PO/ACDS ratio of 2 can be employed to synthesise a suite of carbons with a wide range of porosity. In this scenario, a more environmentally friendly and sustainable activation procedure is allowed, along with easier porosity control, by adjusting the activation temperature.

## 4. Conclusion

We have demonstrated that a less corrosive and less toxic activating agent, potassium oxalate (PO), may be used to produce activated carbons from biomass in a targeted manner. By carefully choosing activation conditions, is it possible to obtain highly microporous activated carbons with surface areas up to 1767 m^2^ g^−1^ and pore volumes up to 0.75 cm^3^ g^−1^. Thus at temperatures of 700–800 °C, the surface area of PO activated carbons is limited to below 1800 m^2^ g^−1^, while surface area of up to 2700 m^2^ g^−1^ may be obtained under similar activation conditions from KOH activation. However, due to their greater microporosity, PO activated carbons have high CO_2_ uptake at low pressure (*i.e.* post-combustion CO_2_ capture conditions). At 25 °C, the PO activated carbons capture up to 1.8 and 5.0 mmol g^−1^ of CO_2_ at 0.15 bar and 1 bar, respectively compared to a maximum of 1.0 and 4.0 mmol g^−1^ for equivalent KOH activated carbons. Unlike what is observed for KOH activation, changes in the amount of PO have no significant influence on the porosity of the PO activated carbons; the activation temperature is the more critical variable in controlling the porosity of the PO activated carbons. As a result, a low amount of PO, coupled with variations in the activation temperature, may be used to generate a suite of carbons with a wide range of targeted porosity.

## Conflicts of interest

There are no conflicts to declare.

## Supplementary Material

RA-012-D2RA02661A-s001
