# Supplementary material for: Rational synthesis of microporous carbons for enhanced post-combustion CO2 capture via non-hydroxide activation of air carbonised biomass
Source: RSC Adv. 2022 Jul 12;12(31):20080–7. doi: 10.1039/d2ra02661a (PMC9275833; doi:10.1039/d2ra02661a)
Supplement: RA-012-D2RA02661A-s001 [file RA-012-D2RA02661A-s001.pdf]

Electronic Supplementary Material (ESI) for RSC Advances.
This journal is © The Royal Society of Chemistry 2022

Supporting Information

Rational synthesis of microporous carbons for enhanced post-combustion CO_2 capture via non-hydroxide activation of air carbonised biomass

Afnan Altwala[a,b], and Robert Mokaya[a*]

[a]School of Chemistry, University of Nottingham, University Park, Nottingham NG7 2RD, U. K.

[b]Department of Chemistry, College of Science Al-Zulfi, Majmaah University, Al-Majmaah, 11952, Saudi Arabia

E-mail: r.mokaya@nottingham.ac.uk (R. Mokaya)

Table S1. Yield and elemental composition of KOH activated carbons derived from ACDS carbon.

Sample	Yield [wt %]	C [%]	H [%]	N [%]	O [%]
Date seed		49.0	7.0	1.6	42.4
ACDS carbon		78.5	4.0	1.2	16.3
ACDS2700	60	82.0	0.9	0.5	16.6
ACDS2800	56	90.0	0.1	0.2	9.7
ACDS4700	54	83.9	0.2	0.3	15.6
ACDS4800	50	84.3	0.1	0.1	15.5
ACDS2700P	58	81.4	1.1	0.3	17.2
ACDS2800P	54	83.3	0.1	0.2	16.4
ACDS4700P	48	82.0	0.7	0.2	17.1
ACDS4800P	46	84.5	0.1	0.3	15.1

Table S2. Textural properties of KOH activated and compactivated carbons derived from ACDS carbon

Sample	Surface area (m^2 g^{-1})	Micropore surface area[a] (m^2 g^{-1})	Pore volume (cm^3 g^{-1})	Micropore volume [b] (cm^3 g^{-1})	Pore size (Å)
ACDS2700	1264	1170 (92)	0.53	0.47 (88)	8.5
ACDS4700	2192	1871 (85)	0.93	0.74 (79)	5.5/8/9/11.5
ACDS2800	2068	1780 (86)	0.88	0.71 (80)	6/9.2/11.7/14.5
ACDS4800	2609	1825 (70)	1.10	0.70 (63)	5.5/8/10/16/20
ACDS2700P	2051	1846 (90)	0.88	0.75 (85)	6/9/11.8
ACDS4700P	2450	1962 (80)	1.05	0.77 (73)	5.8/8/10/11.8/16
ACDS2800P	2357	1889 (80)	1.06	0.75 (71)	6/8.5/12.5/16
ACDS4800P	2738	1793 (65)	1.21	0.69 (57)	5.8/8/10/11.8/16/20

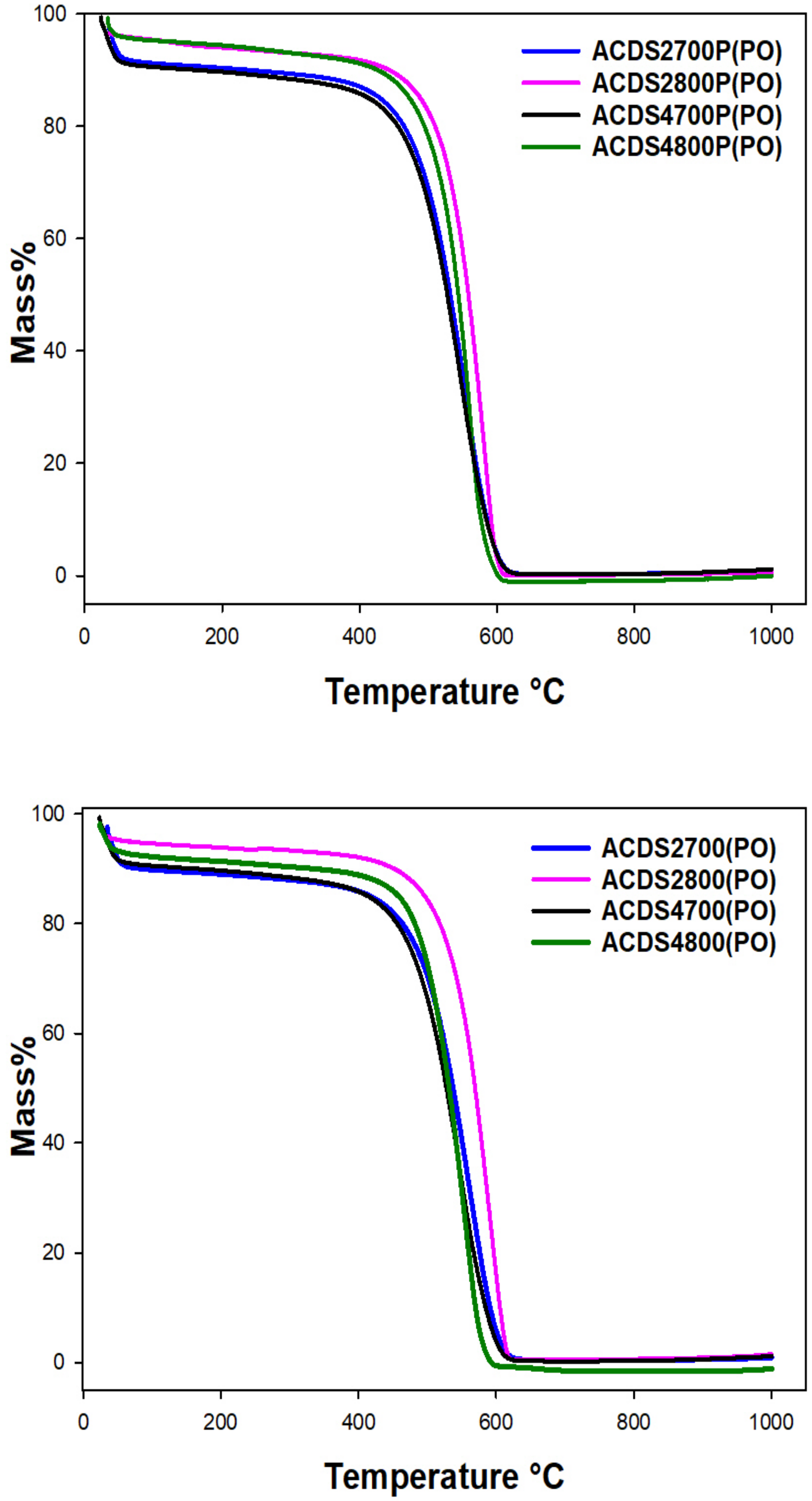

Figure S1. Thermogravimetric analysis curves of PO activated carbons derived from ACDS carbon.

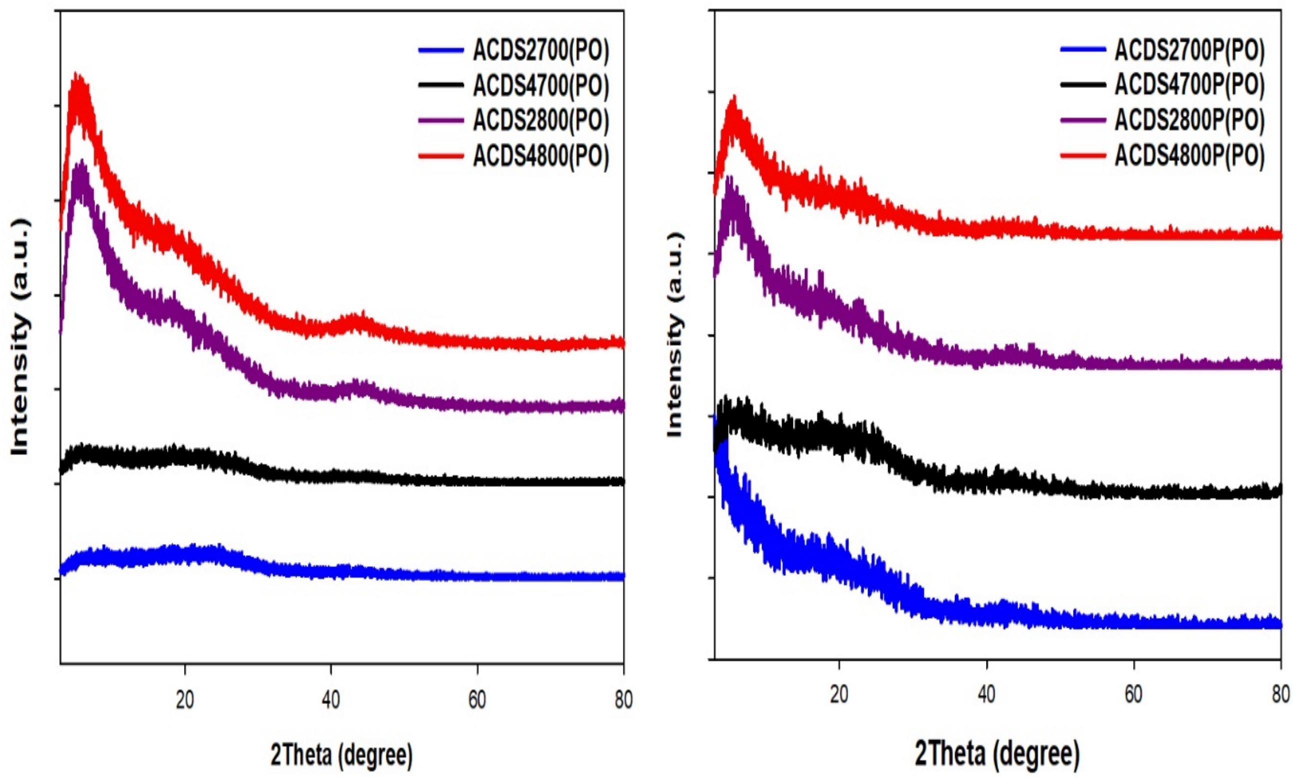

Figure S2. Powder XRD patterns of PO activated carbons derived from ACDS carbon.

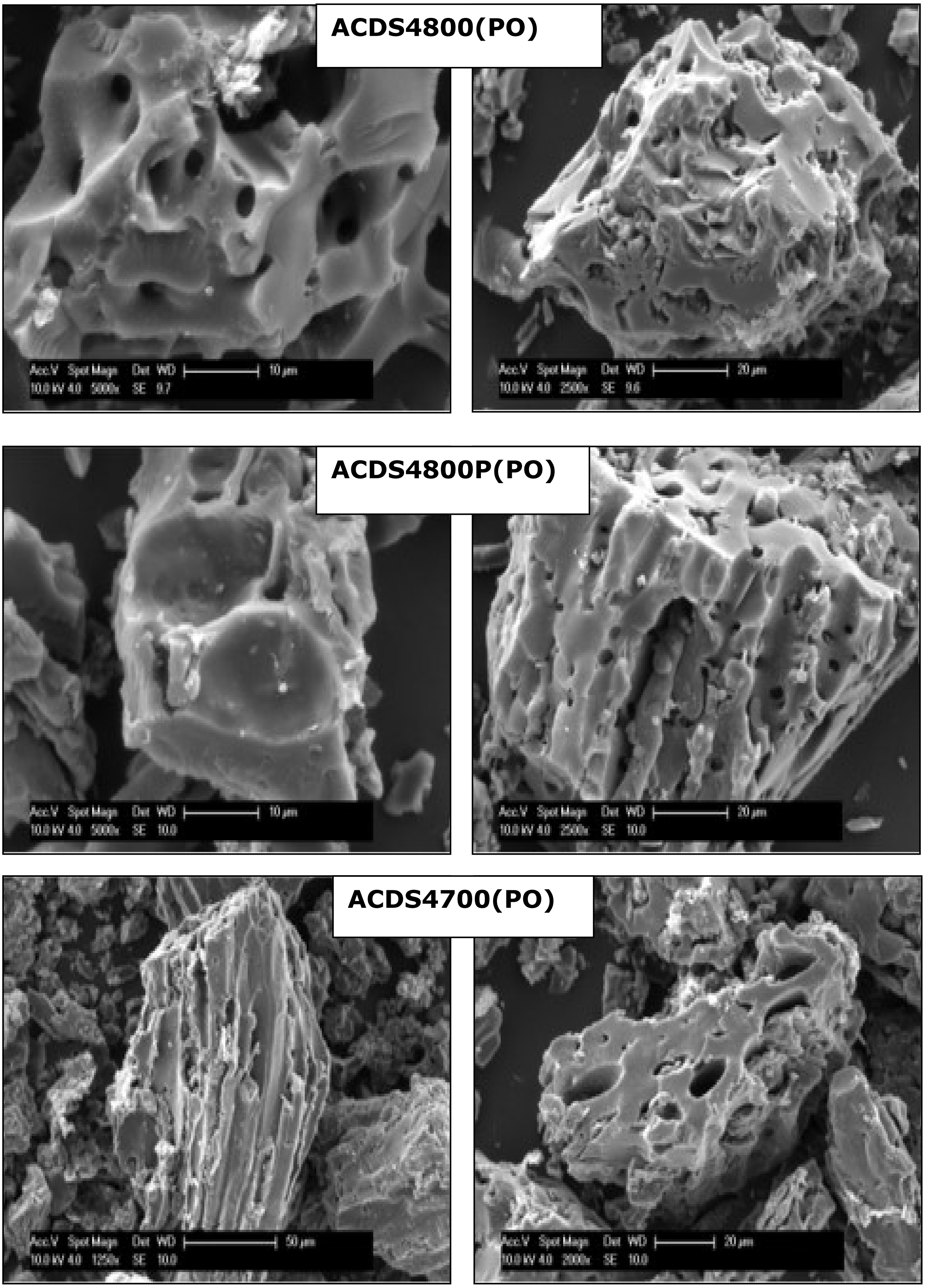

Figure S3. SEM images of PO activated carbons derived from ACDS carbon.

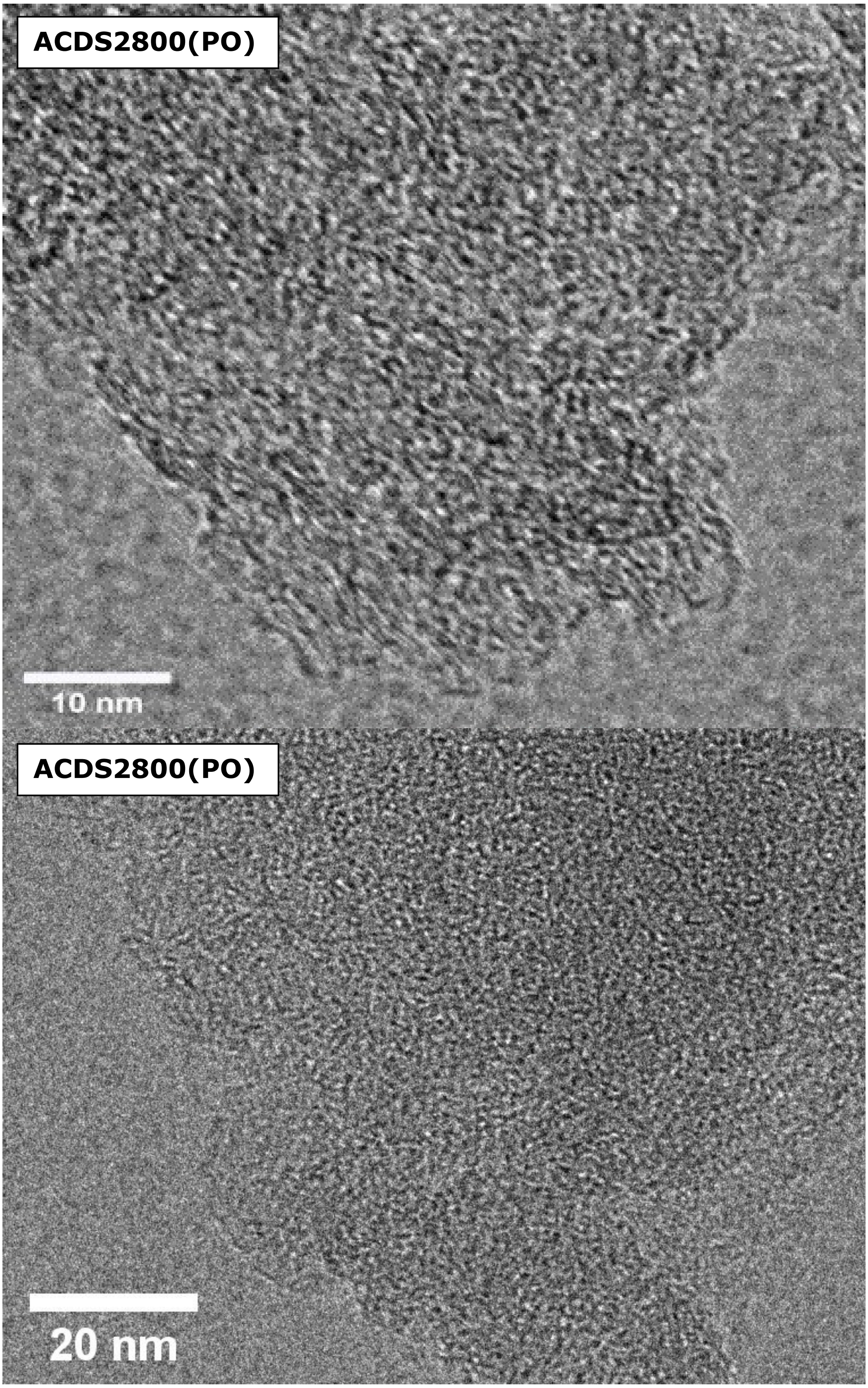

Figure S4. Representative TEM images of sample ACDS2800(PO).

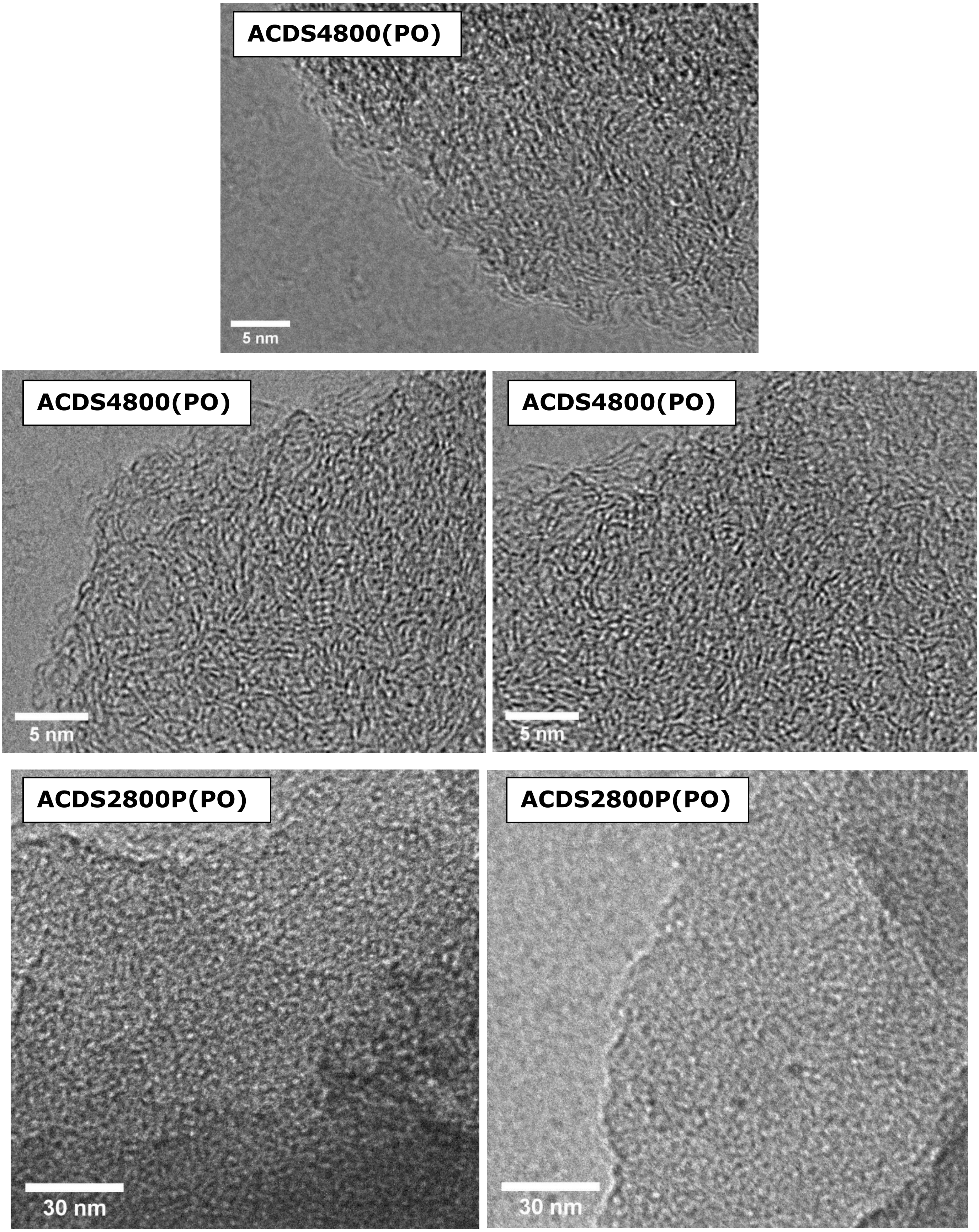

Figure S5. Representative TEM images of sample ACDS4800(PO) and ACDS4800P(PO).

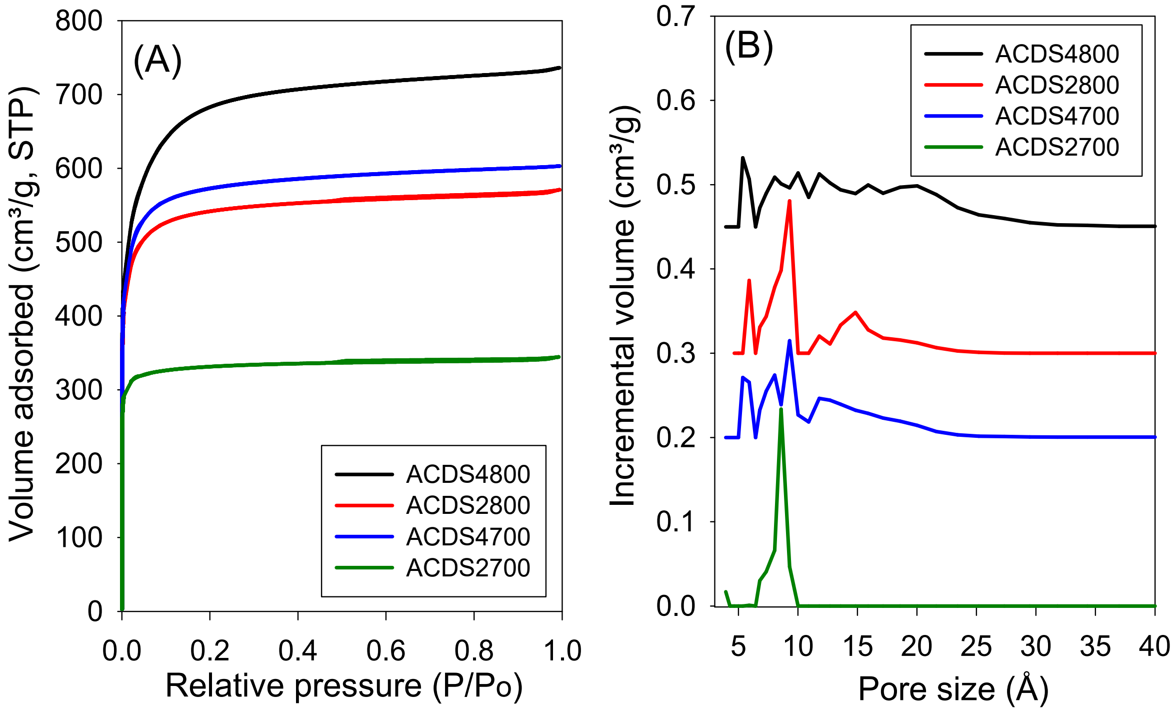

Figure S6. Nitrogen sorption isotherms (A) and pore size distribution curves (B) of KOH activated carbons derived from powdered ACDS carbon.

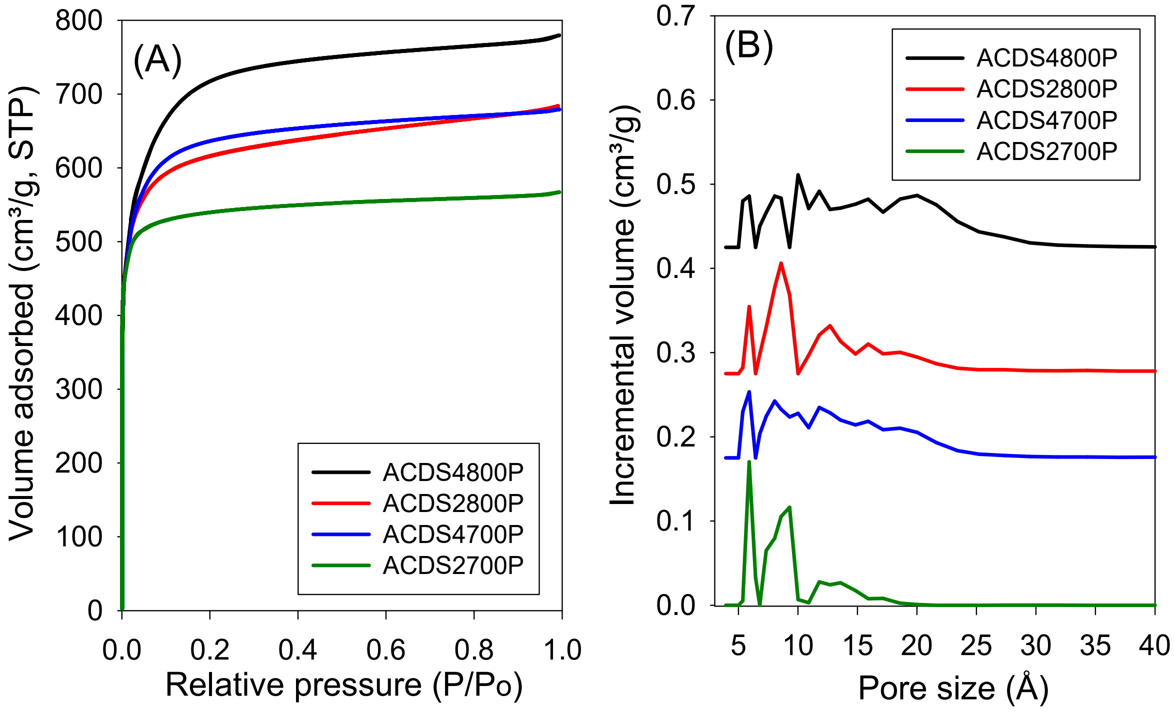

Figure S7. Nitrogen sorption isotherms (A) and pore size distribution curves (B) of KOH compactivated carbons derived from compacted ACDS carbon.

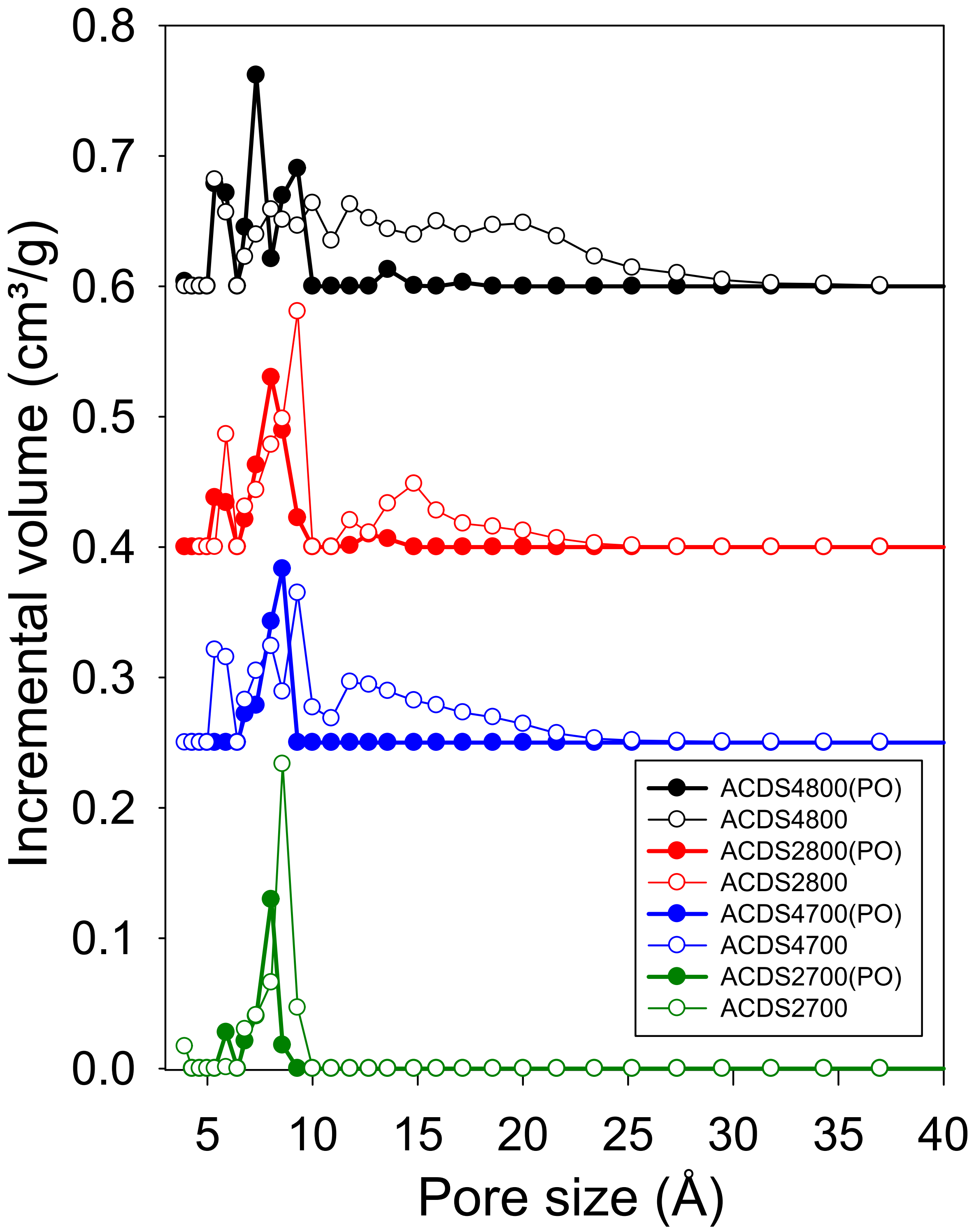

Figure S8. Pore size distribution curves of PO and KOH activated carbons derived from powder ACDS carbon.

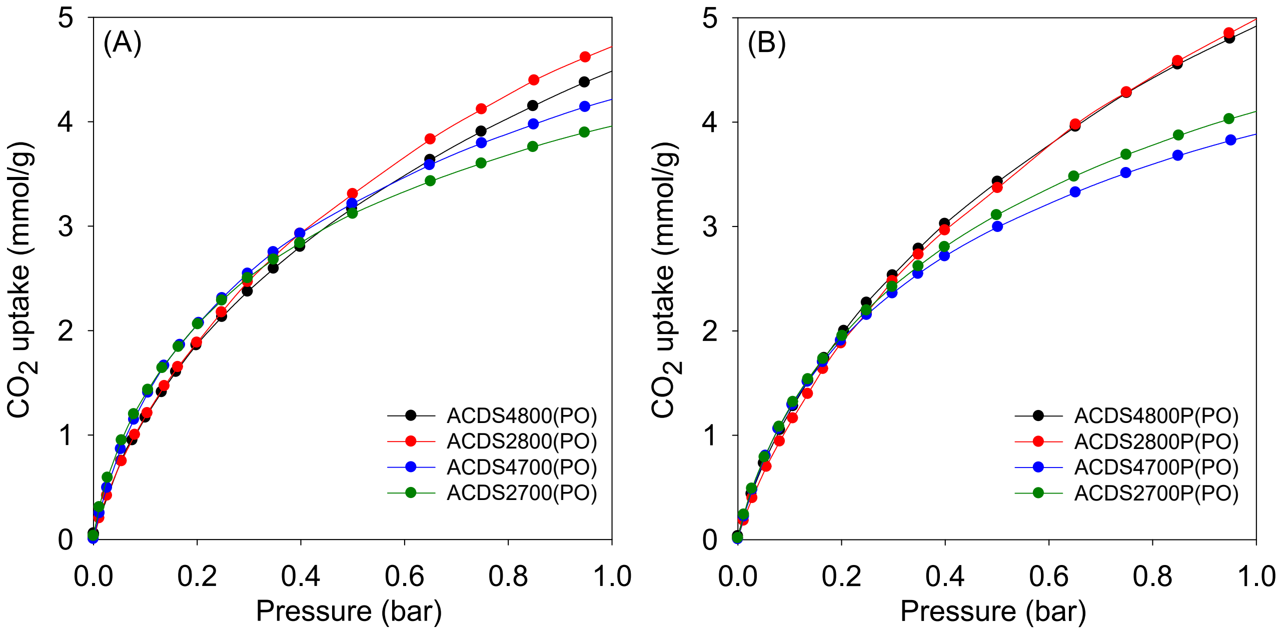

Figure S9. CO_2 uptake of PO activated carbons derived from (A) powdered or (B) compacted ACDS carbon.

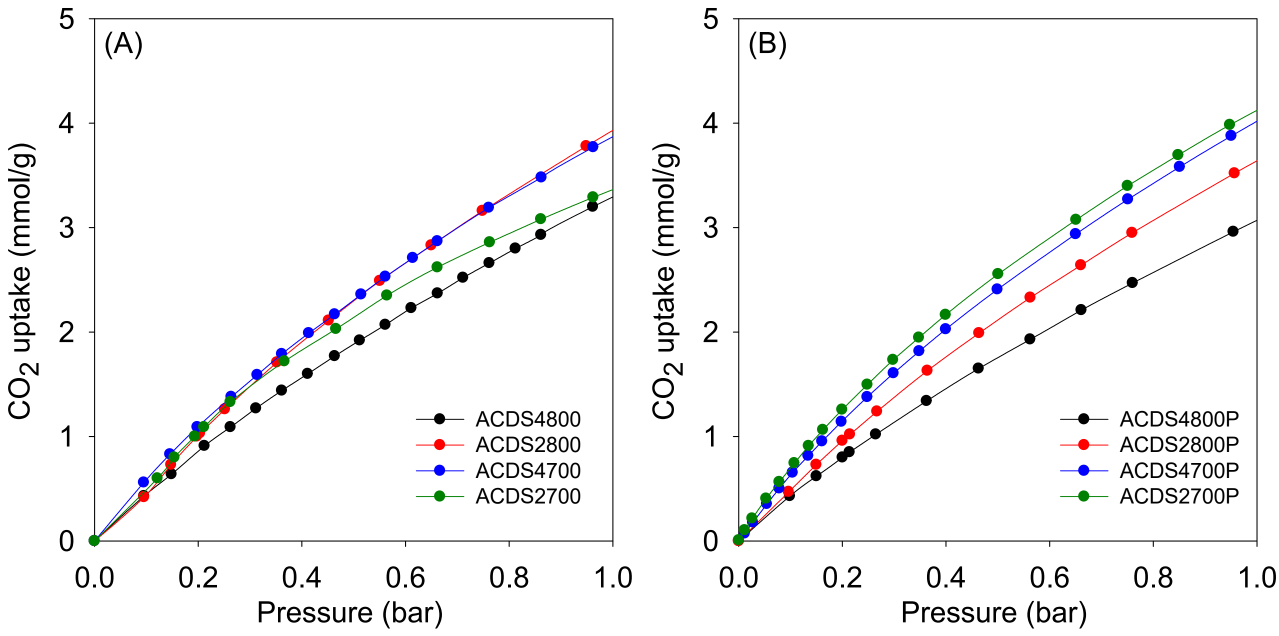

Figure S10. CO_2 uptake of KOH activated carbons derived from (A) powdered or (B) compacted ACDS carbon.
